# Robot-Assisted Laparoscopic Radical Cystectomy and Modified Y-Shaped Ileal Orthotopic Neobladder Reconstruction

**DOI:** 10.3389/fsurg.2022.889536

**Published:** 2022-06-01

**Authors:** Weipu Mao, Shuqiu Chen, Lijie Zhang, Tao Li, Si Sun, Bin Xu, Weidong Zhu, Guangyuan Zhang, Lei Zhang, Jianping Wu, Ming Chen

**Affiliations:** ^1^ Department of Urology, Affiliated Zhongda Hospital of Southeast University, Nanjing, China; ^2^ Surgical Research Center, Institute of Urology, Southeast University Medical School, Nanjing, China; ^3^ Department of Urology, Nanjing Lishui District People’s Hospital, Zhongda Hospital Lishui Branch, Southeast University, Nanjing, China

**Keywords:** bladder cancer, robot-assisted laparoscopic, radical cystectomy, modified Y-shaped ileal orthotopic neobladder, urinary diversion

## Abstract

**Background:**

Orthotopic neobladder reconstruction has become the preferred method of urinary diversion after radical cystectomy in major medical centers. We performed modified Y-shaped ileal orthotopic neobladder reconstruction and presented the functional results and postoperative complications of the modified surgery.

**Methods:**

We included 21 patients with bladder cancer who underwent radical cystectomy at our center between February 2019 and December 2019. All patients underwent robotic-assisted laparoscopic radical cystectomy and lymph node dissection plus modified Y-shaped ileal orthotopic neobladder reconstruction. We collected the demographic and pathological history of the patients, and perioperative and postoperative functional outcomes and postoperative complications were recorded.

**Results:**

All surgeries were successful and no serious postoperative complications occurred. The mean operative time was 321.43 ± 54.75 min, including 101.67 ± 10.88 min required for neobladder reconstruction. Liquid intake was encouraged about 5 days after surgery, stent and catheter were removed after 13.52 ± 3.28 days, and the patients were discharged 1–2 days after removing the catheter. No ureteral anastomotic and neobladder urethral anastomotic strictures occurred. The volume of the neobladder at 1-year post-surgery was 195.24 ± 16.07 mL and the maximum urinary flow rate was 20.64 ± 2.22 mL/s.

**Conclusion:**

We describe the robotic-assisted modified Y-shaped ileal orthotopic neobladder reconstruction performed at our center, which requires a simple suture and short neobladder construction time, minimizes the occurrence of anastomotic stenosis, facilitates smooth patient emptying, and is clinically scalable and applicable.

## Background

According to the 2020 global cancer statistics, bladder cancer (BCa) is the 12th most common cancer with 573,278 new cases, accounting for 3.0% of all new cancers, and 212,536 deaths, approximately 2.1% of all deaths (1). As the second most common malignancy of the genitourinary system, BCa can be categorized into non-muscle-invasive bladder cancer (NMIBC) and muscle-invasive bladder cancer (MIBC). BCa also shows a high recurrence rate and aggressiveness (2, 3). Surgery is the treatment of choice for patients with BCa. Transurethral resection of bladder tumors (TURBT) combined with postoperative intravesical infusion chemotherapy is the main treatment for NMIBC patients (4, 5). For patients with MIBC or high-grade recurrent T1 and/or carcinoma in situ, radical cystectomy (RC) is the best surgical intervention (6, 7). RC can significantly improve tumor control rates and patient survival time.

Urinary diversion after RC is a core treatment for improving patient quality of life and the methods of urinary diversion include an ileal conduit (IC), orthotopic neobladder reconstruction (ONB), and cutaneous ureterostomy (CU) (8, 9). Because ONB can significantly improve patients’ long-term quality of life compared to IC and CU, ONB is now gradually becoming the preferred urinary diversion method after RC in major medical centers (10, 11). Based on the study of Massimo et al (12), our center explored robot-assisted laparoscopic RC and modified Y-shaped ileal ONB in 2019. Here, we report our initial clinical experience, surgical procedures, complications thereof, and oncologic and functional outcomes.

## Patients and Methods

### Patients Selection

A total of 21 patients with bladder cancer who underwent RC at our center between February 2019 and December 2019 were included in the current study. All patients underwent preoperative pelvic magnetic resonance imaging (MRI) or enhanced computed tomography (CT), and preoperative cystoscopic biopsy and the postoperative pathological test confirmed whether it was MIBC or refractory NMIBC. Patients with locally advanced or metastatic disease, stress urinary incontinence, impaired external urethral sphincter, impaired renal function (serum creatinine > 200 μmol/L), severe hepatic impairment, severe small bowel disease, positive prostatic urethral biopsy, and psychiatric disease were excluded. The basic preoperative information of the patients is summarized in Table 1. All patients were followed up until December 2021. The study was approved by the Ethics Committee of the Southeast University Zhongda Hospital (Nanjing, China), and all patients signed written informed consent.

**Table 1:** Preoperative characteristics.

Variable	Result
Patients (*n*)	21
Age (years)	65.76 ± 7.35
Sex (male/female)	21/0
Body mass index (Kg/m^2^)	23.48 ± 2.87
Clinical stage (*n*)	
cTis	1
cT1	13
cT2	5
cT3	2
Serum creatinine, μmol/L	72.65 ± 11.57

*Data for continuous variables are presented as mean ± standard deviation.*

### Surgical Technique

#### Position and Operation Hole

**Figure 1:**
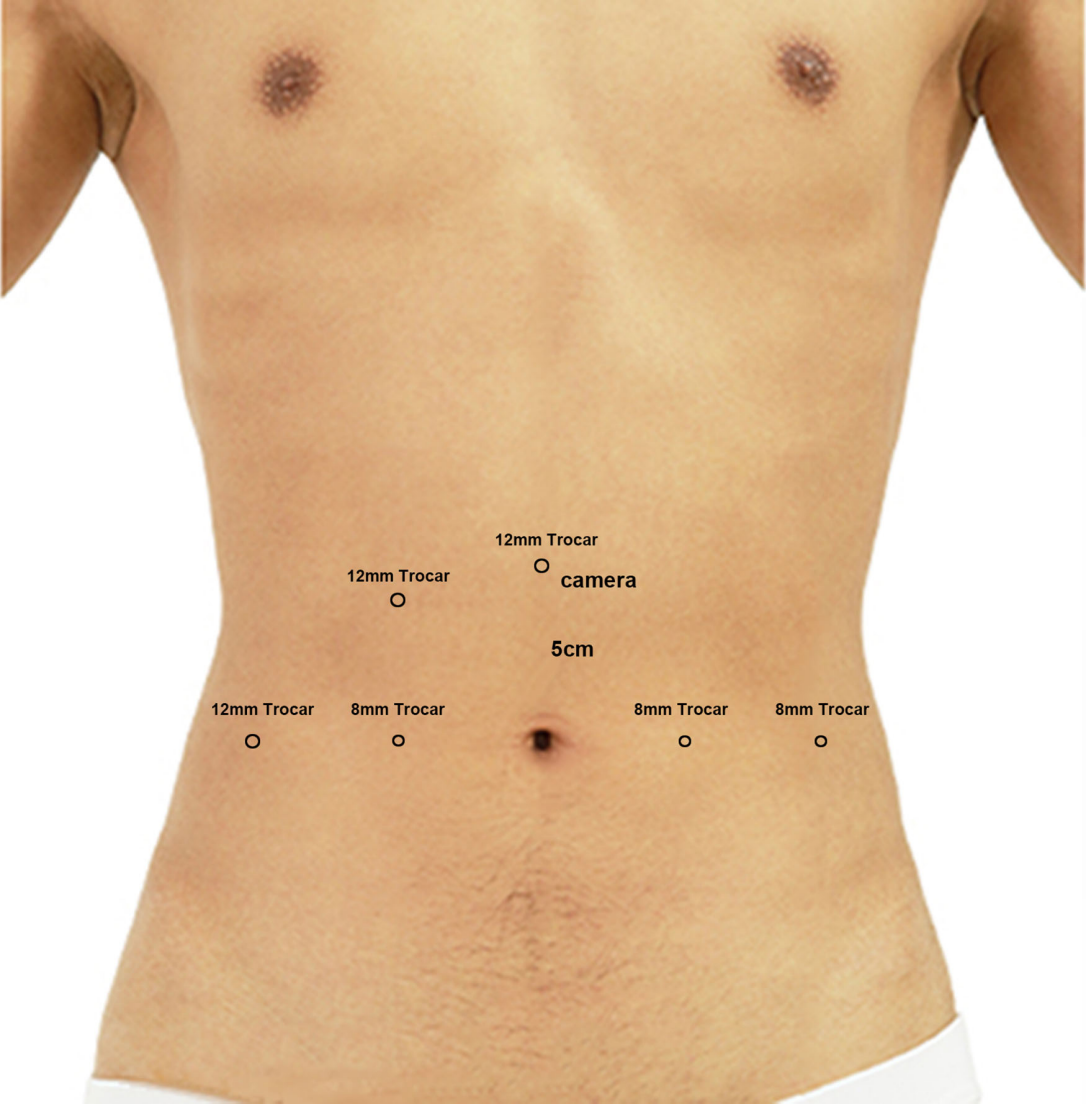
A five-port transperitoneal approach.

The surgery was performed with the patient in the steep Trendelenburg position and a five-port transperitoneal approach was used: the first 12-mm trocar was placed 5 cm above the umbilicus along with the laparoscopic camera; an 8-mm trocar was placed along the lateral aspect of the rectus abdominis muscle where the No. 1 and No. 2 robotic arms were placed; an 8-mm trocar was placed medial to the left iliac spine where the No. 3 robotic arm was placed. In addition, a 12-mm trocar was placed medial to the right iliac spine and at the midpoint of the No. 1 robotic arm and the camera hole where the assistant instruments were placed (Figure 1).

A surgeon (MC) experienced in laparoscopic and robotic-assisted surgery for kidney, prostate and bladder cancers performed all the procedures.

#### “Procedural” Cystectomy

Cystectomy was performed according to our center’s programmed optimization protocol, in the following general order: (1) separation of the right ureter; (2) separation of the bilateral seminal vesicle glands; (3) removal of the right lymph nodes; (4) separation of the right lateral bladder ligament; (5) separation of the left ureter; (6) removal of the left lymph nodes; (7) separation of the left lateral bladder ligament; (8) separation of the retropubic bladder space; (9) opening the pelvic floor and suture ligation of the dorsal deep vein complex (DVC); (10) separation of the lateral prostatic ligament and disconnecting the urethra. After disconnection of the urethra, the prostatic urethral stump is clamped shut using a surgical clip to prevent urine spillage from the bladder. The bladder was then placed in the endobag and finally removed from the incision.

The obturator, internal iliac, external iliac and presacral lymph nodes were cleared during extended pelvic lymph node dissection. Intraoperative frozen sections were used to assess whether the ureteral stump margin and urethral stump margin were positive.

#### Neobladder Preparation and Reconstruction

**Figure 2:**
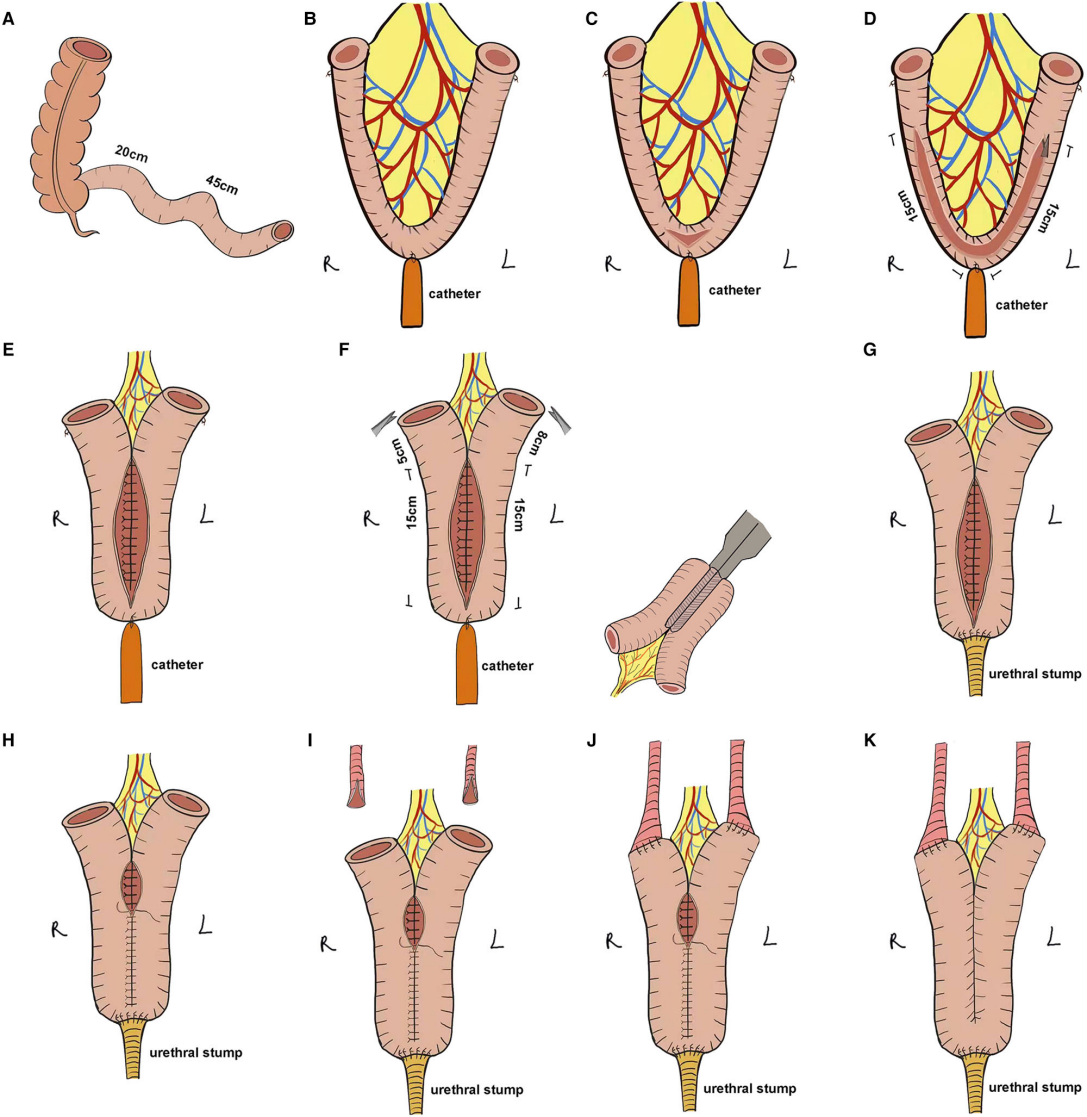
Stepwise configuration of the complete Y-shaped ileal neobladder. (**A**) Approximately 45 cm of ileum was separated for ONB. (**B**) The ileo-urethral anastomosis zone and the ends of the ONB ileum were marked, and the catheter was connected to the ileo-urethral anastomosis area to keep the bowel in tension. (**C**) The intestine between the ileo-urethral anastomosis area and the mesentery was dissected. (**D**) Using the ureteric stent as a ruler, 15 cm of the bowel along each side of the marker was separated. (**E**) The dorsal sides of the left and right intestinal canals were sutured throughout. (**F**) The intestinal canal was truncated at 8 cm from the end of the right intestinal canal suture and 5 cm from the end of the left intestinal canal suture, and the stapler laterally anastomosed the intestine to preserve the intestinal continuity. (**G**) The intestinal wall of the ileo-urethral anastomosis area was anastomosed clockwise to the urethral stump. (**H**) The anterior wall was sutured to the reservoir. (**I**) The ureter was split 1 cm at each end. (**J**) Wallance anastomosis was performed to suture the ends of the ureter to the Y-shaped arms on either side of the reservoir in a continuous manner. (**K**) The remaining anterior wall of the reservoir was anastomosed.

The ileocecal portion was first located and the ONB was performed starting at 20 cm from the ileocecal flap, isolating approximately 45 cm of the ileum for ONB (Figure 2A). The midpoint of the ONB ileum was marked using absorbable sutures as the ileo-urethral anastomosis zone, the ends of the ONB ileum were marked, and a catheter was inserted through the urethra and connected to the ileo-urethral anastomosis zone and then pulled to keep the bowel in tension (Figure 2B). The bowel was dissected with scissors between the ileo-urethral anastomosis zone and the mesentery (Figure 2C), and the ureteral stent was used as a ruler to separate 15 cm of the bowel along each side of the marker (Figure 2D). Subsequently, the dorsal sides of the left and right intestinal canals were sutured throughout (Figure 2E). The intestinal canal was truncated using an ultrasonic knife at 5 cm from the end of the right intestinal canal suture and 8 cm from the end of the left intestinal canal suture. The lateral anastomosis was performed using an Endo-GIA stapler to restore the continuity of the intestine and then, the mesenteric foramen was closed (Figure 2F). The intestinal wall of the ileo-urethral anastomosis area was then anastomosed clockwise to the urethral stump (Figure 2G), and the anterior wall of the anastomotic site was suspended. A catheter and single J stents were inserted, and the ventral sides of the left and right intestinal tubes were sutured successively from the ileo-urethral anastomosis area, and the anterior wall of the reservoir was sutured (Figure 2H).

#### Ureteral Bladder Anastomosis Reconstruction

After splitting the ends of the ureters by 1 cm and anastomosing it with the Y-shaped arms with one stitch (Figure 2I), the single J stent was placed into the ureter up to the renal pelvis, followed by continuous suturing of the ends of the ureters to the Y-shaped arms on both sides of the reservoir using Wallance anastomosis (Figure 2J). Finally, the remaining anterior wall of the reservoir was anastomosed (Figure 2K), and the bilateral single J stent and catheter were exported from the ureter.

### Perioperative and Postoperative Follow-Up

Perioperative and postoperative functional outcomes, postoperative complications, and postoperative voiding were evaluated in the 21 patients, and relevant imaging was performed to determine the volume of the neobladder.

### Statistical Analysis

Data were analyzed using SPSS software (version 24.0). Continuous variables were expressed as mean ± standard deviation.

## Results

The procedure was completed in all the patients without serious complications and conversion to open surgery. The mean operative time was (321.43 ± 54.75) min including the mean time of (101.67 ± 10.88) min required to reconstruct the neobladder. The mean intraoperative blood loss was (129.09 ± 73.55) mL, and only one patient required emergency blood transfusion. The number of intraoperative lymph node dissection was 16.10 ± 6.28, one patient had positive dissected lymph nodes (2/20), and all patients had negative soft tissue surgical margins. The postoperative histopathological staging was slightly altered from that in the preoperative period, with 8 patients being diagnosed with NMIBC (1 with cTis and 7 with cT1) and 13 patients with MIBC (10 with cT2 and 3 with cT3). During the perioperative period, patients resumed fluid intake at a mean of 5.14 ± 0.79 days, and stents and catheters were removed at 13.52 ± 3.28 days. The mean total length of hospital stay for the patients was 14.71 ± 3.20 days (Table 2).

**Table 2:** Perioperative characteristics.

Variable	Result
Total operative time (min)	321.43 ± 54.75
Neobladder time (min)	101.67 ± 10.88
Estimated blood loss (mL)	129.09 ± 73.55
Blood transfusion (*n*)	1
Number of lymph nodes retrieved (*n*)	16.10 ± 6.28
Lymph node metastasis (*n*)	1 (2/20)
Positive soft tissue surgical margins (*n*)	0
Pathological stage (*n*)	
pTis	1
pT1	7
pT2	10
pT3	3
Time to liquid intake (days)	5.14 ± 0.79
Time to stent and catheter removal (days)	13.52 ± 3.28
Hospital stay (days)	14.71 ± 3.20

*Data for continuous variables are presented as mean ± standard deviation.*

**Figure 3:**
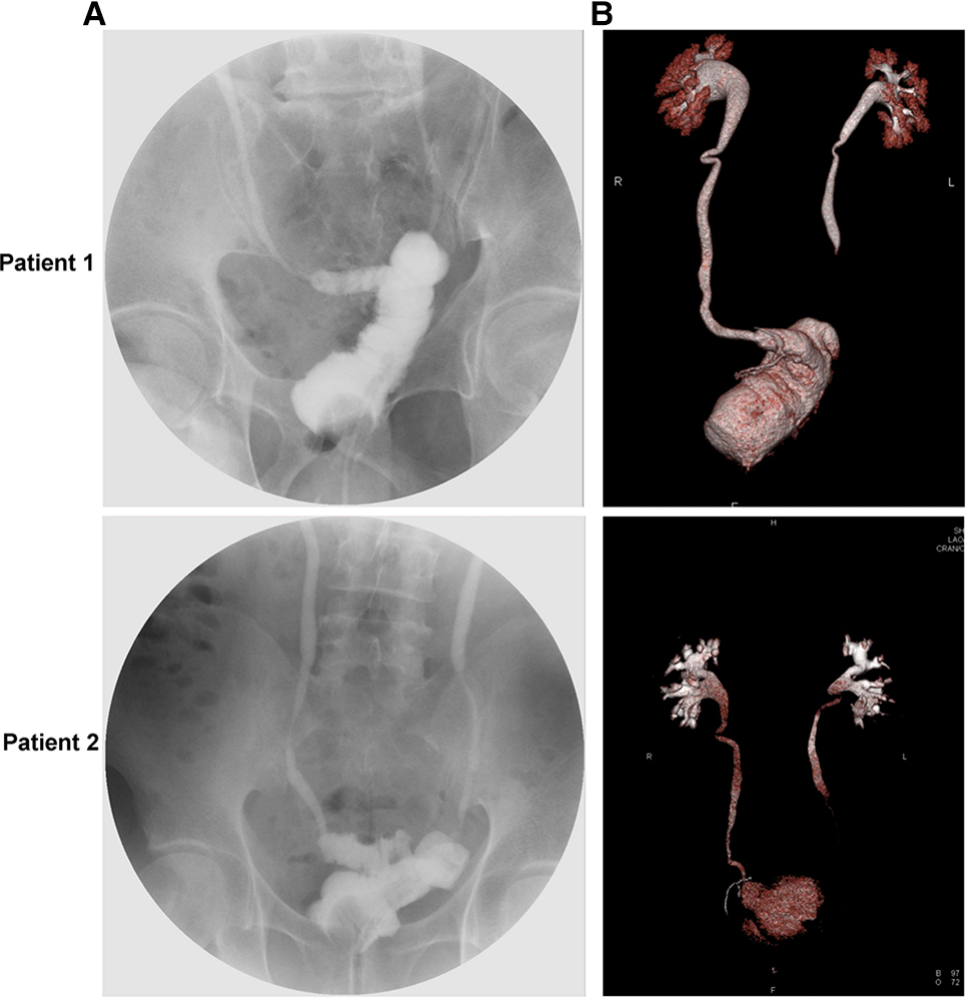
Cystogram and three-dimensional reconstruction images of a representative postoperative Y-shaped neobladder. (**A**) Postoperative cystogram image. (**B**) Postoperative three-dimensional reconstruction image.

All patients were followed up for more than 24 months, and the mean follow-up duration was (29.76 ± 3.65) months, during which no patient died. A total of eight patients developed Clavien-Dindo grade II complications after surgery, including one patient who required blood transfusion therapy, four patients with lung infections, one patient with anemia and two patients with hypoproteinemia (Table 3). Patients voided freely starting two months after surgery, and at the 1-year postoperative follow-up, the mean volume of the patients’ neobladder was 195.24 ± 16.07 mL and the maximum urinary flow rate was 20.64 ± 2.22 mL/s (Figure 3 and Supplementary Figure S1). During the follow-up period, no patient had difficulty in urination and showed recurrence of urethral tumor, neobladder urolithiasis, ureteral anastomotic stricture, and stricture of the neobladder urethral anastomosis. However, one patient developed postoperative incomplete bowel obstruction but the symptoms improved with appropriate treatment (Table 4).

**Table 3:** Postoperative complications.

Variable	Result
Clavien-Dindo classification	
Grade I	0
Grade II	8
Blood transfusion	1
Lung infection	4
Anemia	1
Hypoproteinemia	2
Grade III	0
Grade IV	0

**Table 4:** 1-year postoperative characteristics.

Variable	Result
Difficulty urinating	0
Ureteral anastomotic stricture	0
Neobladder urethral anastomotic stricture	0
Urethral tumor recurrence	0
Neobladder urolithiasis	0
Incomplete intestinal obstruction	1
Neobladder volume (mL)	195.24 ± 16.07
Maximum urine flow rate (mL/s)	20.64 ± 2.22
Follow-up (months)	29.76 ± 3.65
Serum creatinine, μmol/L	69.79 ± 30.60
Overall survival, *n* (%)	21 (100)

*Data for continuous variables are presented as mean ± standard deviation.*

In addition, postoperative daytime and night-time urinary incontinence was recorded for all patients. As shown in Table 5, it was observed that the rates of postoperative daytime and night-time incontinences were 42.86% (9/21) and 61.90% (13/21) at 3 months, 23.81% (5/21) and 38.10% (8/21) at 6 months, and 9.52% (2/21) and 19.05% (4/21) at 1 year, respectively.

**Table 5:** Functional outcomes.

Variable	Patients, *n* (%)		
	3 months	6 months	12 months
Daytime incontinence	9 (42.86)	5 (23.81)	2 (9.52)
Night-time incontinence	13 (61.90)	8 (38.10)	4 (19.05)

*Incontinence was defined as the use of one wet pad or more.*

## Discussion

In the present study, we evaluated the clinical outcomes in 21 patients who underwent modified Y-shaped ileal ONB and found that robot-assisted modified Y-shaped ileal ONB at our center showed satisfactory perioperative and postoperative functional outcomes (Table 6).

**Table 6:** Evaluation of the effectiveness of several robotic-assisted Y-shaped ileal orthotopic neobladder.

Study	Year	N	Neobladder and shape	Surgery Type	Operative time (min)	Neobladder time (min)	Follow up (months)	Afferent limb	Ureteroenteric anastomosis	Complications	Continence outcome
Sim (25)	2015	3	Y shape	RARC	340	NA	NA	Yes	Nesbit	NA	NA
Asimakopoulos (26)	2016	40	Modified Y shape	RARC	315	NA	26.5	No	Wallance	No stricture; 10 (25%) unilat hydronephrosis	Daytime incontinence, 0.0%; Night-time incontinence, 27.5%
Checcucci (28)	2021	45	Modified Y shape	RARC	287	165	14	Yes	Nesbit	2 (4.4%) ureteral anastomotic stricture; 7 (15.5%) acute renal failure	Daytime incontinence, 24.5%; Night-time incontinence, 28.9%
Our study	2022	21	Modified Y shape	RARC	321.43	101.67	29.76	No	Wallance	No stricture	Daytime incontinence, 9.5%; Night-time incontinence, 19.1%

There are several approaches to urinary diversion after RC, depending on the clinical characteristics and surgeon’s considerations. Nevertheless, ONB has become a common choice for patients due to its similarity with the voiding mechanisms (16). Open RC was a surgical approach with a high success rate but the associated uretero-intestinal anastomosis was difficult to visualize due to high surgical difficulty and postoperative complications (17). After the report published by Gill et al. (18) laparoscopy-based RC and ONB have been further developed and matured. Robot-assisted laparoscopic radical cystectomy and neobladder reconstruction have also recently gained popularity due to the introduction of the da Vinci® SI system, and ONB reconstruction is performed at an increasing number of centers (18–20). In some medical centers, the proportion of ONB after RC has increased to 50%–90%. In 2007, Hautmann et al. (11) retrospectively summarized a total of 7,129 reports on postoperative ureteral diversion methods after RC from multiple medical centers worldwide, of which ONB accounted for 46.9%, IC accounted for 32.7%, and controlled ureteral diversion skin stoma accounted for 7.6%. However, in 2015, Hugen (21) reported that 75% of patients at the largest medical center in the United States receive ONB, with only a minority of patients (less than 15%) opting for IC.

ONB can be classified as gastric ONB, ileal ONB, ileocolonic ONB, and sigmoid ONB depending on the segment of the GI tract used (22). Due to the convenience of ileal retrieval and low pressure of the neobladder after detubularization, which is conducive to the protection of the patient’s renal function, the ileum is currently most widely used to construct the neobladder (22, 23). The Camey II ileal neobladder was a modification of Camey I ileal neobladder reconstruction (24). The Camey II ileal neobladder reconstruction method separates a 65-cm long section of the ileum, arranges the bowel in a U-shape, and performs a ureter-ileal anastomosis using the Le Due method. The Camey II ileal neobladder reconstruction achieves a low-pressure reservoir that better protects the upper urinary tract function and improves daytime urinary control. Mrini et al. (25) reported that after the Camey II procedure, patients showed a postoperative ureteral reflux rate of 6.8% and ureteral anastomotic stricture rate of 8.6%, with 64.2% of patients achieving daytime urinary control immediately post-surgery and 80% daytime urinary control at 3 months post-surgery.

In 2012, Massimo and colleagues modified the Camey II procedure by decannulating a 45-cm section of the ileum and arranging it in a vertical Y-shape (12). The advantage, therein, was that the intercepted bowel was shorter and the volume of the constructed storage bladder was relatively smaller, which, together with the Y-shaped bladder, was more conducive to postoperative bladder emptying and avoidance of residual urine, thereby reducing metabolic acidosis. Massimo found that all 237 patients who received the Camey II modification were able to empty their bladders postoperatively, with daytime and night-time urinary control rates of 93.5% and 83.9%, respectively (12).

However, the incidences of ureteral neobladder stricture, neobladder urethral stenosis, and neobladder urolithiasis in Massimo’s modified approach were 3.3%, 6.3%, and 16.0%, respectively. After exploring the causes of complications in Massimo’s Y-shaped neobladder approach, we further improved it. Firstly, we used the da Vinci robotic operating system, which utilizes the advantages of minimally invasive robotic surgery. Secondly, we used absorbable thread in suturing the neobladder instead of staples, which, too, reduced the incidence of neobladder urolithiasis. Thirdly, we split the end of the ureter and sutured it to the intestinal break of the reservoir using the Wallance anastomosis to reduce the incidence of ureteral neobladder stenosis. Finally, we reserved a longer urethral stump and the anterior wall of the anastomotic site was draped during the neobladder urethral anastomosis, which reduced the incidence of neobladder urethral stricture and improved urinary control in patients postoperatively.

To date, there were several different ileal neobladder procedures, including Studer ileal neobladder, Camey II ileal neobladder, M/W-shaped ileal neobladder, Kock ileal neobladder, Mainz ileal neobladder, and Le Bag ileal neobladder (10, 11, 26). The Y-shaped neobladder was first described by Fontana in 2004 (27), who demonstrated that the ileal Y-shaped neobladder showed good functional outcomes comparable to the mainly diffused ileal neobladder, and several subsequent studies have confirmed the feasibility of ileal Y-shaped neobladder surgery (13–15, 28, 29). Compared to other ileal neobladder procedures, our modified Y-shaped ileal neobladder requires only one bowel fold and simple suturing. Combined with the advantages of minimally invasive robotic surgery, the construction time of the neobladder is relatively short, which greatly reduces the surgery time and facilitates the patient’s postoperative recovery. The modified Y-shaped ileal neobladder requires a shorter bowel, which is more conducive to postoperative bladder emptying and avoiding residual urine and impaired renal function, and none of the patients experienced acute kidney injury. Unlike the Y-shaped neobladder described by Fontana, we used absorbable thread instead of staples in suturing the neobladder, and none of the patients developed neobladder urolithiasis postoperatively. In addition, unlike other Y-shaped neobladder approaches, we drew bilateral single J stents from the urethra, which facilitated fixation and reduced the number of perforations in the abdominal wall, which facilitated the patient’s postoperative recovery.

Although the volume of the modified Y-shaped ileal neobladder immediately after surgery was lower, after 1 year, patients achieved a volume of 200 mL and daytime and nocturnal urinary control rates of 90.5% and 81.0%, respectively. In addition, we used the Wallance anastomosis for the ureteral neobladder anastomosis, which increased the area of the ureteral ileum and greatly reduced the incidence of ureteral neobladder stenosis. Also, the sides of the Y-shaped bowel of the neobladder were connected to the bilateral ureters on both sides, the ureters did not need to be displaced again, and both sides of the intestine could play an anti-urinary reflux effect.

There were a few limitations of this study. Since this study was conducted only at our center, the number of included cases was small, and the patients were followed up for just over two years. A multicenter study with a longer follow-up period needs to be conducted. In addition, one patient in this study presented with an emergency blood transfusion. A retrospective review of the patient’s clinicopathological data revealed that the patient was older and had a large tumor size and abundant blood supply, which may have caused the transfusion and required us to develop a reasonable indication. Moreover, several medical centers do not have a robotic operating system due to gaps across the regional medical centers, which may cause differential effects that need to be evaluated with further research on this technology.

## Conclusions

We report the clinical efficacy of a robot-assisted modified Y-shaped ileal ONB at our center. Although the follow-up period was less, preliminary results were encouraging, with a low rate of postoperative complications and better postoperative functional outcomes. This also suggested that robot-assisted laparoscopic modified Y-shaped orthotopic ileal ONB is a scalable option for ONB.

## Data Availability

The raw data supporting the conclusions of this article will be made available by the authors, without undue reservation.
