# Supplementary figures and images for: Robot-Assisted Laparoscopic Radical Cystectomy and Modified Y-Shaped Ileal Orthotopic Neobladder Reconstruction
Source: Front Surg. 2022 Jun 1;9:889536. doi: 10.3389/fsurg.2022.889536 (PMC9198462; doi:10.3389/fsurg.2022.889536)

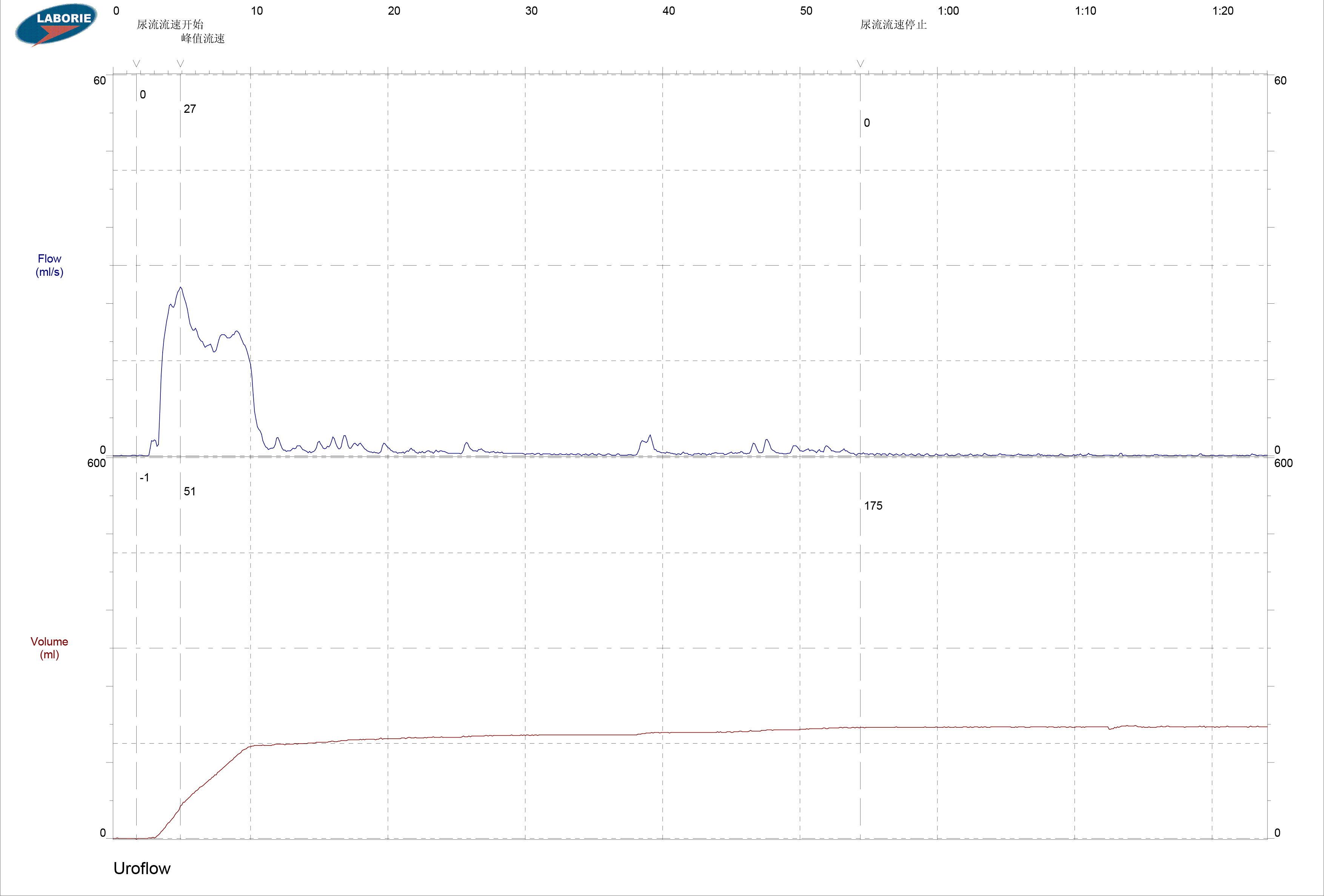

Supplement: Supplementary file 1 [file Image_1_v1.jpeg]
